# Mental health and academic experiences among U.S. college students during the COVID-19 pandemic

**DOI:** 10.3389/fpsyg.2023.1166960

**Published:** 2023-04-27

**Authors:** Michael E. Roberts, Elizabeth A. Bell, Jillian L. Meyer

**Affiliations:** Department of Psychology and Neuroscience, DePauw University, Greencastle, IN, United States

**Keywords:** mental health, college students, COVID-19, academic performance, remote learning, perceived stress, anxiety, depression

## Abstract

When the COVID-19 pandemic began, U.S. college students reported increased anxiety and depression. This study examines mental health among U.S college students during the subsequent 2020–2021 academic year by surveying students at the end of the fall 2020 and the spring 2021 semesters. Our data provide cross-sectional snapshots and longitudinal changes. Both surveys included the PSS, GAD-7, PHQ-8, questions about students’ academic experiences and sense of belonging in online, in-person, and hybrid classes, and additional questions regarding behaviors, living circumstances, and demographics. The spring 2021 study included a larger, stratified sample of eight demographic groups, and we added scales to examine relationships between mental health and students’ perceptions of their universities’ COVID-19 policies. Our results show higher-than-normal frequencies of mental health struggles throughout the 2020–2021 academic year, and these were substantially higher for female college students, but by spring 2021, the levels did not vary substantially by race/ethnicity, living circumstances, vaccination status, or perceptions of university COVID-19 policies. Mental health struggles inversely correlated with scales of academic and non-academic experiences, but the struggles positively correlated with time on social media. In both semesters, students reported more positive experiences with in-person classes, though all class types were rated higher in the spring semester, indicating improvements in college students’ course experiences as the pandemic continued. Furthermore, our longitudinal data indicate the persistence of mental health struggles across semesters. Overall, these studies show factors that contributed to mental health challenges among college students as the pandemic continued.

## Introduction

Before the COVID-19 pandemic, 1 in 5 U.S. college students reported “overwhelming anxiety,” and 1 in 6 reported depression severe enough to impact daily functioning (American College Health Association, 2018), but the pandemic led to worse mental health among this cohort. For example, Wang et al. (2020b) surveyed over 2000 U.S. college students in May 2020, and found that 48% reported moderate-to-severe depression and 38% reported moderate-to-severe anxiety. A longitudinal study at Dartmouth (Huckins et al., 2020) conducted before and during the first academic term of the pandemic found higher depression, anxiety, and sedentary time, and the anxiety and depression were correlated with more COVID-19 news. Furthermore, pandemic-related stressors may have exacerbated symptoms for students who were already experiencing depression (Greaney et al., 2021).

These pandemic-related mental health impacts among college students appear to be a global phenomenon. A survey of French college students in April and May of 2020 indicated that 16% had severe depression, 28% had a high level of anxiety, 22% had severe distress, and 11% had suicidal thoughts (Wathelet et al., 2020). In May, 2020, 15% of college students in Bangladesh reported high levels of severe depression, and 18% reported severe anxiety (Islam et al., 2020). In a sample of college students in Saudi Arabia between March and June of 2020, 35% reported moderate to extreme levels of anxiety (Khoshaim et al., 2020). A survey of Chinese college students in February and March 2020 found that 44% reported increased stress levels, 17% had anxiety symptoms, and both measures of mental health were correlated with more frequent reading of the news during that time period (Wang et al., 2020a). A meta-analysis by Li et al. (2021) found high levels of depression and anxiety among college students across 27 studies, and percentages of symptoms were higher among non-Chinese students and higher after March 1, 2020.

Studies have identified several factors correlated with pandemic mental health impacts. Females have generally reported higher levels of psychological distress (e.g., Li et al., 2021), more time on news was correlated with negative mental health outcomes in Wathelet et al. (2020) and Huckins et al. (2020), and social media usage was correlated with greater stress for female and male college students (Prowse et al., 2021). A change to living at home with family correlated with worse mental health among samples of college students in some studies (e.g., Islam et al., 2020), but living with family was a protective factor in another study (Cao et al., 2020), and some findings suggest that negative effects may be due to financial pressures placed on families during the pandemic (Islam et al., 2020).

College students’ anxiety and depression levels may have remained high throughout the 2020–2021 academic year because their academic and social experiences were still disrupted in myriad ways. For example, a follow-up (Mack et al., 2021) to the longitudinal Dartmouth study found that as the pandemic continued in spring 2020, anxiety and depression among college students were still higher than previous academic terms and were correlated with the internet search term “COVID fatigue.” The transition to remote learning was challenging for many students (e.g., Aristovnik et al., 2020; Wang et al., 2020b; Prowse et al., 2021), and many universities continued to predominantly offer remote and hybrid classes during subsequent semesters and adopted campus policies that reduced the number and perhaps quality of in-person interactions among students.

Our research objective was to better ascertain the mental health of U.S. college students as the pandemic continued, and we sought to determine potential differentiating factors of depression, anxiety, and perceived stress among students. During the fall 2020 semester, we conducted a survey study of U.S. college students. We hypothesized that students living on-campus would have better mental health scores than students living off-campus, and we hypothesized that participants who spent more time on social media platforms such as Facebook, Instagram, Twitter, and Tiktok would have worse mental health scores. In regards to academic experiences, we hypothesized that participants taking in-person classes would have a higher sense of belonging to their class community than participants taking online classes. During spring 2021, we conducted a follow-up study for further cross-sectional as well as longitudinal examinations of mental health among college students. We believed that factors such as universities’ COVID-19 mitigation policies as well as individuals’ perceived risks of contracting COVID may influence mental health changes as the pandemic continued, so we added questions to assess these factors. With the new cross-sectional data, we revisited the hypotheses from the fall study, and we also hypothesized that mental health scores would improve for the longitudinal participants from Survey 1 to Survey 2, that those scores would correlate with perceived risk of contracting COVID-19 and with vaccination status, and that those scores and the quality of students’ social lives would correlate with universities’ COVID-19 mitigation policies.

## Method

Using Prolific^1^ for a U.S. nationwide sample, we conducted a mental health survey of 279 college students in the fall 2020 semester and then, in the spring 2021 semester, conducted a second survey of 399 students, including 120 participants from the fall sample. Both surveys collected measures of perceived stress, anxiety, depression, academic and non-academic experiences, living situation, and extent of news and social media usage. Our surveys also asked about students’ academic experiences and sense of belonging in three respective class formats – in-person, virtual, and hybrid. In the spring 2021 study, we refined the questions regarding students’ academic and non-academic experiences, and we intentionally collected a stratified sample with equal representation in several race/ethnicity and gender categories.

### Survey 1 (fall 2020)

#### Participants

Participants were recruited via Prolific (see footnote 1), an international survey site that pays participants to partake in the surveys. The site is similar to Amazon Mechanical Turk (Palan and Schitter, 2018) but has additional filtering mechanisms, ethical compensation, and has been repeatedly shown to provide higher quality data (Peer et al., 2021; Douglas et al., 2023) and recommended as a better source for collecting data from participants online (Newman et al., 2021). Like Mechanical Turk, Prolific provides access to convenience samples that are likely more diverse than laboratory studies, and it has been used to collect diverse samples in studies with the same mental health measures during the pandemic (e.g., Groarke et al., 2020; Armour et al., 2021; Sams et al., 2021). Table 1 presents the participant demographics. Compared to the demographics by race of U.S. undergraduate enrollment (American Council on Education, n.d.), the proportion of participants identifying as Asian appeared to be over-represented, while the proportions of participants identifying as Black and Hispanic were underrepresented. Our recruitment posting included filters to only allow participants who were pursuing a Bachelor’s Degree in the United States and were 18 years of age or older. The survey took about 7.5 min to complete, and participants were awarded $1.30 upon completion. Of the 279 responses, 10 participants were excluded for failing one or more of the random attention checks, and 10 participants were excluded because they were not currently pursuing a Bachelor’s degree, despite the Prolific filter.

**Table 1:** Sample size and means/SDs for key scales and sociodemographic characteristics.

Variable	Category	Fall 2020	Spring 2021
Gender	Women	135	205
	Men	114	169
	Other	10	4
Race/ethnicity	Caucasian	136	101
	Black/African American	17	64
	Hispanic/Latino	16	57
	Asian/Asian-American	53	94
PSS	Women	23.79(7.23)	23.01(7.49)
	Men	20.29(8.36)	19.94(8.41)
	White/Caucasian	23.15(7.54)	20.99(7.13)
	Black/African American	21.89(8.74)	21.40(9.78)
	Hispanic/Latino	24.39(6.75)	19.10(9.49)
	Asian/Asian-American	21.15(8.23)	21.14(8.15)
GAD	Women	11.30(5.50)	9.47(5.76)
	Men	8.07(5.31)	7.93(5.41)
	White/Caucasian	10.76(5.39)	9.11(5.35)
	Black/African American	7.44(6.14)	8.02(5.38)
	Hispanic/Latino	10.67(5.26)	9.25(6.25)
	Asian/Asian-American	8.72(5.60)	8.20(5.54)
PHQ	Women	10.87(6.14)	11.10(6.41)
	Men	8.54(6.11)	8.69(6.19)
	White/Caucasian	10.41(6.10)	9.67(6.17)
	Black/African American	9.44(6.35)	10.28(6.47)
	Hispanic/Latino	11.78(5.68)	11.28(6.97)
	Asian/Asian-American	9.00(6.39)	9.05(6.31)
Place of residence	Within Commuting Distance	147	199
	Outside of Commuting Distance	58	81
	On Campus	40	98
Socioeconomic status	Low (less than $25,000)	-	58
	Medium-Low ($25,000-50,000)	-	83
	Medium ($50,000-100,000)	-	134
	Medium-high ($100,000-200,000)	-	70
	High (More than $200,000)	-	32
Working status	Not-working	80	157
	Part-Time	83	111
	Full-Time	43	40
Class-year	First Year	30	56
	Sophomore	58	84
	Junior	85	123
	Senior	62	98
	Fifth-Year	13	16
School type	Private (Less than 5,000 students)	–	36
	Private (5,000–15,000 students)	–	31
	Private (More than 15,000 students)	–	20
	Public (Less than 5,000 students)	–	12
	Public (5,000–15,000 students)	–	90
	Public (More than 15,000 students)	–	187

#### Materials

##### Perceived stress scale

This consists of 11 questions on 5-point Likert scales ranging from *never* to *very often*, where higher scores indicate higher feelings of stress (Cohen et al., 1983).

##### Generalized anxiety disorder test

This consists of seven questions on 4-point Likert scales ranging from not at all to *nearly every day*, where higher scores indicate higher feelings of anxiety (Spitzer et al., 2006).

##### Patient health questionnaire

This consists of nine questions on 4-point Likert scales ranging from *not at all* to *nearly every day*, where higher scores indicate higher feelings of depression (Kroenke et al., 2009).

##### Additional questions

Additional Likert scale questions asked about perceived support levels, amount of time spent on social media and news media, and academic experiences and sense of community in in-person, remote, and hybrid college classes during the fall 2020 semester. There were also four attention check questions that were Likert-scale based and distributed throughout the survey. Additional forced-choice questions asked about gender identity, job status, location, etc. When we created the survey, we included a wide variety of questions based on students’ expressed interests for data analyses in an undergraduate course on Research Methods in Psychology. However, the three authors examined a constrained set of hypotheses for this survey and in conducting the follow up study. A full set of the survey questions can be found in the Supplementary material.

#### Procedure

Data were collected with a Google Forms survey^2^. This study was reviewed and approved by the DePauw University IRB Board. Participants were recruited on the Prolific website (see footnote 1) and provided informed consent *via* the Google Form after they were told the purpose of the study. Responses were received between November 3, 2020 and November 11, 2020.

### Survey 2 (spring 2021)

#### Participants

For the spring survey we received 399 total responses with 120 of them being the same participants from the fall. The Prolific restrictions were the same as the fall survey with the addition of an age restriction of 18–22 years old, and we posted the survey eight times with separate demographic restrictions to obtain nearly 50 participants from each demographic combination of two gender categories (Female and Male) and four race/ethnicity categories (Black/African American, Asian/Asian-American, Hispanic/Latino, White). Survey completion averaged 9.13 min. Most participants received $1.43 upon completion of the survey, but in an attempt to recruit a representative sample after low initial group participation rates, we re-posted surveys with appropriate filters to collect data from additional Hispanic females, Black males, and Black females with $2.00 in compensation. Twenty-one participants were excluded for failing one or more attention checks. Table 1 shows the participant demographics. For analyses that compared demographic groups, we excluded participants who did not fit into our two gender categories or four race/ethnicity categories, leaving 313 participants.

#### Materials

A full set of the survey questions can be found in the Supplementary material. A major addition to this follow-up study was an examination of the effects of different university COVID-19 policies on student mental health and academic success. We conducted informal interviews with students at several colleges and used recurring themes to create the subscales below with high face validity, and our analyses in the Results sections suggest high reliability for all but one of the scales.

##### COVID-19 monitoring scale

This assessed the extent to which participants’ universities were monitoring their COVID-related behaviors during the spring 2021 semester. It included three questions on 5-point Likert scales.

##### Social quality on campus scale

This assessed the perceived social quality of a participant’s campus experience during the spring 2021 semester. It included five questions on 5-point Likert scales.

##### Perceived effectiveness of COVID-19 policies scale

This assessed the perceived effectiveness of their university’s handling of COVID-19 on campus during the spring 2021 semester. It included three questions on 5-point Likert scales.

##### Academic experience scale

This assessed how participants felt about their academic experience as a whole during the spring 2021 semester. It included three questions on 5-point Likert scales.

##### Non-academic experience scale

This assessed how participants felt about their non-academic experiences during the spring 2021 semester. It included four questions on 5-point Likert scales.

##### Additional questions

Additional Likert scale questions asked about perceived support levels, how much time participants spend on social media and news media, whether they had received a COVID-19 vaccination, and, if not, their enthusiasm about receiving a vaccination. There were also four attention check questions that were Likert-scale based and distributed throughout the survey. Additional forced-choice questions asked about gender identity, job status, etc. We also used the PSS, GAD-7, and PHQ-8 surveys described in the Study 1 materials.

#### Procedure

The data were collected with a Google Forms survey (see footnote 2) with Likert scales and forced-choice questions when appropriate. This study was reviewed and approved by the DePauw University IRB Board. Participants were recruited on the Prolific website (see footnote 1) and provided informed consent via the Google Form after they were told the purpose of the study. Responses were received between April 22, 2021 and May 13, 2021.

## Results

### Fall 2020 mental health among college students

Table 1 presents demographic data as well as the means and standard deviations for the PHQ-8, GAD-7, and PSS. Cronbach’s α scores indicate reliability for these samples (fall PHQ-8 = 0.90; fall GAD-7 = 0.91; fall PSS = 0.91). We note that normality assumptions were not met in some cases (e.g., PHQ-8, GAD-7, PSS scores), but we report parametric tests, which are considered appropriate with large sample sizes and provide more direct comparison with most of the published literature in this area; however, nonparametric tests of these data show similar results (e.g., regarding statistical significance for the mental health score comparisons). Female students (n = 135) scored higher than males (n = 114) on all three measures (PHQ-8: *t*(247) = 2.98, *p* = 0.003, *d* = 0.38; GAD-7: *t*(247) = 4.70, *p* < 0.001, *d* = 0.60; PSS: *t*(247) = 3.54, *p* < 0.001, *d* = 0.45). These scores were near clinical thresholds. Between groups ANOVAs showed that students’ living situations – on-campus, off-campus within commuting distance, and off-campus beyond commuting distance – did not correspond to significant differences on the PHQ-8 (*p* = 0.70), GAD-7 (*p* = 0.44), or PSS (*p* = 0.92). Amount of time spent on social media correlated with PHQ-8 [*r*(257) = 0.18], GAD-7 [*r*(257) = 0.28], and PSS [*r*(257) = 0.24]. Amount of time spent on news media correlated with PHQ-8 [*r*(257) = 0.17] and GAD-7 [*r*(257) = 0.14] but not with PSS [*r*(257) = 0.09].

### Fall 2020 academic experiences among college students

Table 2 shows participants’ reported academic experiences and sense of belonging for three class formats. For the subset of 95 participants who reported experiences in all three class formats, a repeated measures ANOVA comparing Academic Experience by class format did not show a difference, *F*(2, 188) = 1.96, *p* = 0.14. However, for the subset of 96 participants who reported their sense of community in all class formats, a repeated measures ANOVA showed a difference, *F*(2, 190) = 17.99, *p* < 0.001, with LSD post-hoc tests indicating that in-person classes (M = 2.71, SD = 1.06) led to a significantly more belonging than hybrid classes (M = 2.29, SD = 0.94), which was significantly more than fully online classes (M = 2.01, SD = 1.09). The subsample’s means and standard deviations across conditions were consistent with the full sample in Table 2.

**Table 2:** Participants’ sense of community and academic experience by semester and class format.

Variable	Fall		Spring	
	*M* (SD)	*n*	*M* (SD)	*n*
*Sense of community:*				
In person	2.74 (1.06)	106	3.47 (1.22)	163
Hybrid	2.28 (0.99)	130	2.83 (1.08)	185
Online	1.88 (1.14)	248	2.28 (1.20)	365
*Academic experience:*				
In Person	2.55 (0.95)	105	3.56 (1.19)	159
Hybrid	2.63 (0.98)	130	3.32 (1.01)	185
Online	2.63 (1.25)	248	3.06 (1.27)	365

### Spring 2021 and longitudinal mental health among college students

Table 1 presents demographics and the means and standard deviations for the PHQ-8, GAD-7, and PSS. Cronbach’s α scores indicate reliability for these samples (spring PHQ-8 = 0.89; spring GAD-7 = 0.92; spring PSS = 0.90). Female students scored higher than males on all three measures (PHQ-8: *t*(372) = 3.67, *p* < 0.001, *d* = 0.38; GAD-7: *t*(372) = 2.63, *p* = 0.009, *d* = 0.27; PSS: *t*(372) = 3.62, *p* < 0.001, *d* = 0.39). There was a similar count of vaccinated (n = 180) and not vaccinated (n = 197) participants, but independent samples t-tests showed no differences in their scores on the PHQ-8 (*p* = 0.87), GAD-7 (*p* = 0.26), or PSS (*p* = 0.63); however, the question did not differentiate between partly and fully vaccinated, and given the timing of the survey and the age group, it is possible that many of the “vaccinated” individuals had recently received only their first of two doses. Between groups ANOVAs showed that students’ living situations – on-campus, off-campus within commuting distance, and off-campus beyond commuting distance – did not correspond to differences on the PHQ-8 (*p* = 0.60), GAD-7 (*p* = 0.99), or PSS (*p* = 0.48). For the subset of 120 participants with longitudinal data, repeated measures ANOVAs showed no differences in PHQ-8 or GAD-7 across semesters, but there was mixed evidence for a decrease among PSS scores (*p* = 0.07 for a two-way paired-samples *t*; *p* = 0.01 for a Wilcoxon signed rank test). Cronbach’s 𝛼 scores also indicate reliability for these longitudinal samples (fall PHQ-8 = 0.89; spring PHQ-8 = 0.88; fall GAD-7 = 0.89; spring GAD-7 = 0.92; fall PSS = 0.88; spring PSS = 0.90).

Reliable responses were obtained for the Monitoring (Cronbach’s 𝛼=0.66), Academic Experience (Cronbach’s 𝛼=0.75), and Non-Academic Experience (Cronbach’s 𝛼=0.74) scales. The Perceived Effectiveness scale was reliable (Cronbach’s 𝛼=0.55) after removing the question about university COVID-19 policies affecting stress (Cronbach’s 𝛼=0.35 with the item included), but responses to the Social Quality scale were surprisingly inconsistent (Cronbach’s 𝛼=0.27) across the five items; therefore, we did not include the Social Quality scale in analyses.

The perceived effectiveness of university COVID-19 policies was positively correlated with Academic Experience, *r*(376) = 0.15, *p* = 0.004, and Non-Academic experience, *r*(376) = 0.18, *p* < 0.001. As shown in Table 3, the Academic and Non-Academic Experience scales were inversely correlated with PHQ-8, GAD-7, and PSS scores, whereas academic workload was positively correlated with the GAD-7 and PSS. Time on social media was positively correlated with the PHQ-8, GAD-7, and PSS, whereas time on news media and the perceived risk of COVID-19 were not correlated with any mental health measures.

**Table 3:** Mental health and COVID-19 policies Spring 2021.

COVID-19 Policy (*N* = 378)	Pearson correlations		
	PSS	GAD	PHQ
Monitoring_1_	0.001	−0.01	−0.03
Perceived effectiveness_3_	0.003	−0.03	−0.04
Academic experience_4_	−0.42**	−0.30**	−0.28**
Academic workload_1_	0.18**	0.11*	0.08
Non-academic experience_1_	−0.45**	−0.29**	−0.33**
Perceived risk of COVID_1_	0.07	0.03	0.03
Time on social media_2_	0.15**	0.12*	0.17**
Time on news_1_	−0.06	0.02	−0.04

**Correlation is significant at the 0.01 level (2-tailed).

*Correlation is significant at the 0.05 level (2-tailed).

*N* = 378_1_, 377_2_, 376_3_, 375_4_.

### Spring 2021 and longitudinal academic experiences among college students

Table 2 shows the means and standard deviations for academic experience and sense of belonging in respective class formats (in-person, remote, and hybrid). For the 140 participants who reported experiences in all three class formats, a repeated measures ANOVA comparing Academic Experience by class format was significant, *F*(2, 278) = 10.83, *p* = 0.001, with LSD post-hoc tests indicating that in-person classes (M = 3.51, SD = 1.22) led to a significantly better experience than hybrid classes (M = 3.26, SD = 1.05), which were significantly better than fully online classes (M = 2.91, SD = 1.21). The respective means and standard deviations across conditions were consistent with the full sample in Table 2. Sense of community effects were even more pronounced for the 146 participants who reported scores for all three class formats, *F*(2, 290) = 62.47, *p* < 0.001, with LSD post-hoc tests indicating that in-person classes (M = 3.45, SD = 1.25) led to a significantly better sense of community than hybrid classes (M = 2.74, SD = 1.04) which were significantly better than fully online classes (M = 2.18, SD = 1.18). Again, this subset was consistent with the overall sample in Table 2.

For the subset of 120 participants with longitudinal data, repeated measures ANOVAs showed increases in class experience and sense of belonging for all class formats (all at *p = *0.001 or below). A comprehensive 2 (fall or spring semester) X 3 (Class Format: In-person, Online, or Hybrid) repeated measures ANOVA, which only included 29 participants who experienced all three class formats in both semesters, showed a comparable main effect for semester for both measures and a main effect for class format in which in-person participants reported higher sense of belonging than hybrid, which was higher than online.

### Spring 2021 subset analyses by race/ethnicity and school type

After filtering to participants who chose one designation for race/ethnicity, between groups ANOVAs indicated no differences by race/ethnicity for PSS (*p* = 0.85), GAD-7 (*p* = 0.43), or PHQ-8 (*p* = 0.20). Table 4 reports the means for these demographic subsets in the longitudinal data. There were no differences by race/ethnicity for the Academic Experience (*p* = 0.40) and Non-Academic Experience (*p* = 0.22) scales. There was a significant difference in time spent on social media, *F*(3, 311) = 9.53, *p* < 0.001, with Black and Hispanic participants tending to spend more time than Asian and White participants, but there was no difference in time spent on news (*p* = 0.88). There were no differences in ratings on the Monitoring scale (*p* = 0.39), Perceived Effectiveness scale (*p* = 0.39), or satisfaction with their college’s COVID-19 handling for the spring (*p* = 0.17).

**Table 4:** Longitudinal participants’ mental health Fall 2020 to Spring 2021.

		Fall 2020				Spring 2021			
Category			Mean Scores				Mean Scores		
		*n*	PSS	GAD-7	PHQ-8	n	PSS	GAD-7	PHQ-8
Women (*n* = 58)	White/Caucasian	31	24.3 (5.95)	12.52 (4.90)	11.19 (5.91)	31	22.39 (4.80)	11.13 (5.50)	10.84 (5.56)
	Black/African American	4	20.2 (4.35)	3.75 (1.50)	6.75 (2.06)	4	16.25 (2.50)	2.75 (1.71)	3.75 (2.06)
	Hispanic/Latino	3	27.6 (9.45)	8.00 (2.65)	12.00 (7.94)	3	22.33 (4.16)	7.00 (4.36)	10.00 (7.00)
	Asian/Asian-American	20	20.8 (7.29)	9.10 (5.04)	8.90 (6.32)	20	20.40 (6.64)	8.55 (5.72)	9.75 (7.08)
Men (*n* = 46)	White/Caucasian	32	19.9 (6.29)	8.56 (3.94)	7.69 (5.32)	32	18.59 (5.24)	7.28 (4.56)	7.47 (5.66)
	Black/African American	4	24.0 (10.86)	5.75 (6.65)	8.75 (7.63)	4	18.00 (8.98)	4.75 (5.74)	7.00 (7.44)
	Hispanic/Latino	1	28.0 ()	10.00 ()	14.00 ()	1	17.00 ()	4.00 ()	7.00 ()
	Asian/Asian-American	9	20.8 (10.59)	6.56 (4.93)	8.89 (6.11)	9	23.89 (4.96)	13.00 (4.09)	13.00 (5.63)

After filtering to participants who attended private (n = 87) or public colleges (n = 289), between groups ANOVAs indicated no differences by college type for PSS (*p* = 0.40), GAD-7 (*p* = 0.66), PHQ-8 (*p* = 0.25), the Academic Experience (*p* = 0.18) and Non-Academic Experience (*p* = 0.12) scales, and the time spent on social media (*p* = 0.73) and news (*p* = 0.08). Students at private colleges reported higher ratings on the Monitoring scale (*F*(1, 374) = 9.37, *p* = 0.002) and marginally higher ratings on the Perceived Effectiveness scale (*F*(1, 374) = 3.79, *p* = 0.052); however, there was no difference in satisfaction with their college’s COVID-19 handling (*p* = 0.36).

### Longitudinal relationships between mental health and academic experience

We conducted three hierarchical regression analyses. The first evaluated the effects of several factors on spring academic experience and academic confidence, respectively. We added fall academic confidence in the first block (Model I), fall PHQ-8, GAD-7, and PSS in the second block (Model II), and spring PHQ-8, GAD-7, PSS, and Non-Academic Experience scores in the third block (Model III). The respective models explained 13, 14, and 47% of the variance for spring academic experience, and 26, 27, and 46% of the variance for spring academic confidence. In both analyses, among the mental health scores, only the fall PSS scores were a significant predictor (β = 0.25, *p* = 0.04 for academic experience; β = 0.26, *p* = 0.03 for academic confidence; however, these positive β values compare to negative β values for the other non-significant mental health measures, including spring PSS), but spring Non-Academic Experience was a critical predictor (β = 0.57, *p* < 0.001 for academic experience; β = 0.40, p < 0.001 for academic confidence).

The second regression analysis predicted spring Non-Academic Experience. We added fall academic confidence in the first block (Model I), fall PHQ-8, GAD-7, and PSS in the second block (Model II), and spring PHQ-8, GAD-7, and PSS in the third block (Model III). The respective models explained 5, 14, and 20% of the variance. Fall PSS scores were the only significant predictor among the mental health measures (β = −0.34, *p* = 0.02).

## Discussion

Numerous studies have shown that mental health among college students and young adults has been negatively impacted during the early months of the COVID-19 pandemic (e.g., Huckins et al., 2020; Wang et al., 2020b). By surveying students during the fall 2020 and spring 2021 semesters, we assessed whether those effects persisted, and we tested associations with academic and non-academic factors. Our fall data indicated near-clinical levels of depression, anxiety, and perceived stress as measured by the PHQ-8, GAD-7, and PSS, and the spring cross-sectional and longitudinal data show that high levels of depression and anxiety persisted, while there was mixed evidence for a decrease in PSS scores. In contrast, Zheng et al. (2022) found reduced PSS and PHQ-9 scores by spring 2021, but their study compared to an earlier spring 2020 timepoint. Consistent with our hypotheses and previous research (e.g., Li et al., 2021; Rettie and Daniels, 2021), female college students had significantly worse mental health during these time periods. No differences in mental health measures were found between race/ethnicity groups. We also found support for our hypotheses that more time on social media would correlate with worse mental health; in contrast, time spent on news media correlated with mental health impacts in the fall 2020 sample but not the spring 2021 sample.

Our research also assessed college students’ academic experiences during the pandemic and the extent to which those experiences and universities’ COVID-19 mitigation policies correlated with mental health measures. As we hypothesized, the fall 2020 sample indicated a clear rank order for sense of belonging in college classes, with the highest for in-person, then hybrid, then remote. There was no significant difference in the reported quality of class experience across these formats; however, the larger spring 2021 sample showed significant differences in sense of belonging and academic experience across class formats, with the same ordering as in the fall. The subset of participants with longitudinal data showed the same pattern, though their ratings for both sense of belonging and class experiences improved from the fall 2020 to spring 2021 semester. Unfortunately, those improvements were clearly insufficient by themselves to address the pandemic mental health impacts among college students. A regression analysis indicated that spring academic experience was significantly influenced by spring non-academic experiences but not by measures of mental health in the fall or spring. However, spring non-academic experiences were partly predicted by fall perceived stress scores, indicating some potential indirect roles for how mental health impacted subsequent academic experiences. Follow-up research may help to delineate the most impactful components of that non-academic experience. Related work by Cahuas et al. (2023) used a detailed assessment of perceived social support and found that support from family was a strong predictor of quality of life among college students during the pandemic. Our non-academic experiences scale only had one item about social support, but it had the possible benefit of including several items about satisfaction with the campus living situation and community.

Aside from large gender differences, we found few differentiators of mental health impacts from demographics or living circumstances. Across race/ethnicity groups, there were no significant differences in levels of anxiety, depression, and perceived stress, or in academic experiences or perceptions of university’s handling of COVID-19 pandemic. This appears consistent with Charles et al. (2021), who found that White students reported larger mental health impacts than Black students in the early weeks of the pandemic but showed similar impacts at a later timepoint, and it is also consistent with Liu et al. (2022) who found no differences in PSS responses by ethnicity in late fall 2020. Similarly, in our spring 2021 sample, there were no significant differences between students at private and public universities in terms of mental health impacts or reported levels of academic and non-academic experiences, though students at private colleges reported higher levels of monitoring and marginally higher levels of effectiveness. Contrary to our hypotheses, students who lived on campus did not have higher mental health scores than those living off campus. Additionally, perceived differences in campus COVID-19 policies did not correlate with mental health scores, and neither vaccination status nor students’ perceived risk of contracting COVID-19 correlated with mental health scores.

Some of our results may reflect survey limitations. The fall 2020 survey was conducted shortly before the U.S. presidential election, which may have impacted mental health scores as well as news media coverage. In the spring 2021 sample, our question about vaccination status did not differentiate between students who were fully vaccinated and students who had recently had a first dose, and there may have been a large number of college students in that latter group due to the timing of vaccine availability for different demographics. The demographically stratified sample is a clear strength of our spring 2021 data, but its representativeness is nonetheless limited by comparatively fewer numbers of Black male participants. Finally, the social quality scale in the spring 2021 sample did not show internal reliability, so our results could not address whether that was a differentiating factor in students’ mental health across colleges.

We believe our research makes a valuable contribution toward understanding the trajectory of college students’ mental health at different timepoints of the COVID-19 pandemic Our findings suggest that under circumstances like a pandemic that may greatly disrupt universities’ normal functioning, universities should nonetheless prioritize in-person learning as much as possible, as sense of belonging was much higher in that class format, and such non-academic experiences significantly inversely correlated with scores of depression, anxiety, and perceived stress. Moreover, non-academic experience was a significant predictor of academic experience and academic confidence in the current semester. Even if a university can continue with in-person classes in such circumstances, it should also provide options for students to take leaves of absence or pursue remediation among increased responsibilities (e.g., Lv et al., 2022), as a study by Brown et al. (2023) suggests that many students’ educational plans changed during the pandemic, and results by Lee et al. (2021) suggest first generation students were especially likely to take time off. It is worth noting that some universities have continued to provide online-learning options since the pandemic, and the seemingly negative implications of this on social connection might be something to consider with future courses offered. Our findings also have a *potential* recommendation for individual college students: consider decreasing your social media usage to see if there are mental health benefits. Of course, the consistent association that we find between social media and mental health across semesters is only correlational, much like related research that shows associations between internet addiction and mental health among Czech students (Gavurova et al., 2022), but it may be worth trying habit changes as well as practicing effective coping strategies (Awoke et al., 2021).

Overall, our results show a persistent mental health decline among U.S. college students during the 2020–2021 academic year. According to students, the overall academic experience improved during that time period, but even in spring 2021, in-person classes were significantly better than hybrid and remote classes. The qualities of academic and non-academic experiences were inversely correlated with mental health scores, and our longitudinal analyses indicate some connections between these constructs across semesters, with perceived stress in fall 2020 associated with non-academic experiences in spring 2021, which were in turn associated with academic experiences during that semester. We anticipate that the strength of these relationships will change in subsequent academic years as universities return to many of their pre-pandemic norms in terms of academic and social experiences.

## Data availability statement

The raw data supporting the conclusions of this article will be made available by the authors, without undue reservation.

## Ethics statement

The studies involving human participants were reviewed and approved by DePauw University Institutional Review Board. The patients/participants provided their written informed consent to participate in this study.

## Author contributions

MR, EB, and JM contributed to the conception and design of the fall and spring studies. MR performed the statistical analyses. EB and JM created the tables. MR wrote the first draft of the manuscript and performed the statistical analyses. All authors contributed to the manuscript revision and read and approved the submitted version.

## Funding

The J. William and Katherine C. Asher Endowed Research Fund grant S-21-i provided support for this study.

## Conflict of interest

The authors declare that the research was conducted in the absence of any commercial or financial relationships that could be construed as a potential conflict of interest.

## Publisher’s note

All claims expressed in this article are solely those of the authors and do not necessarily represent those of their affiliated organizations, or those of the publisher, the editors and the reviewers. Any product that may be evaluated in this article, or claim that may be made by its manufacturer, is not guaranteed or endorsed by the publisher.

## Footnotes

^1^ https://www.prolific.co/

^2^ https://docs.google.com/forms

## References

[ref1] American College Health Association (2018). American College Health Association-National College Health Assessment II: Undergraduate Student Reference Group Data Report Fall 2018. Silver Spring, MD: American College Health Association.

[ref001] American Council on Education (n.d.). Race and Ethnicity of U.S. Undergrauates. Retrieved March 30, 2023, from https://www.equityinhighered.org/indicators/enrollment-in-undergraduate-education/race-and-ethnicity-of-u-s-undergraduates/

[ref2] Aristovnik A. Keržič D. Ravšelj D. Tomaževič N. Umek L. (2020). Impacts of the COVID-19 pandemic on life of higher education students: a global perspective. Sustainability 12:8438. doi: 10.3390/su12208438 PMC8634691 34869802

[ref3] Armour C. McGlinchey E. Butter S. McAloney-Kocaman K. McPherson K. E. (2021). The COVID-19 psychological wellbeing study: understanding the longitudinal psychosocial impact of the COVID-19 pandemic in the UK; a methodological overview paper. J. Psychopathol. Behav. Assess. 43, 174–190. doi: 10.1007/s10862-020-09841-4, PMID: 33169046 PMC7641483

[ref4] Awoke M. Mamo G. Abdu S. Terefe B. (2021). Perceived stress and coping strategies among undergraduate health science students of Jimma University amid the COVID-19 outbreak: online cross-sectional survey. Front. Psychol. 12:639955. doi: 10.3389/fpsyg.2021.639955, PMID: 33859594 PMC8042268

[ref5] Brown M. L. Trotter C. E. Huang W. Contreras Castro K. DeMuth W. D. Bing E. G. (2023). COVID-19 and mental health among college students in the southwestern United States. J. Am. Coll. Heal. 1–8, 1–8. doi: 10.1080/07448481.2022.2153601, PMID: 36701420

[ref6] Cahuas A. Marenus M. W. Kumaravel V. Murray A. Friedman K. Ottensoser H.. (2023). Perceived social support and COVID-19 impact on quality of life in college students: an observational study. Ann. Med. 55, 136–145. doi: 10.1080/07853890.2022.2154943, PMID: 36519501 PMC9762801

[ref7] Cao W. Fang Z. Hou G. Han M. Xu X. Dong J.. (2020). The psychological impact of the COVID-19 epidemic on college students in China. Psychiatry Res. 287:112934. doi: 10.1016/j.psychres.2020.112934 32229390 PMC7102633

[ref8] Charles N. E. Strong S. J. Burns L. C. Bullerjahn M. R. Serafine K. M. (2021). Increased mood disorder symptoms, perceived stress, and alcohol use among college students during the COVID-19 pandemic. Psychiatry Res. 296:113706. doi: 10.1016/j.psychres.2021.113706 33482422 PMC7781902

[ref9] Cohen S. Kamarck T. Mermelstein R. (1983). A global measure of perceived stress. J. Health Soc. Behav. 24, 385–396. doi: 10.2307/2136404 6668417

[ref10] Douglas B. D. Ewell P. J. Brauer M. (2023). Data quality in online human-subjects research: comparisons between MTurk, prolific, CloudResearch, Qualtrics, and SONA. PLoS One 18:e0279720. doi: 10.1371/journal.pone.0279720, PMID: 36917576 PMC10013894

[ref11] Gavurova B. Khouri S. Ivankova V. Rigelsky M. Mudarri T. (2022). Internet addiction, symptoms of anxiety, depressive symptoms, stress among higher education students during the COVID-19 pandemic. Front. Public Health 10:893845. doi: 10.3389/fpubh.2022.893845, PMID: 35774570 PMC9237380

[ref13] Greaney J. L. Darling A. M. Turner J. R. Saunders E. F. H. Almeida D. M. Mogle J. (2021). COVID-19-related daily stress processes in college-aged adults: examining the role of depressive symptom severity. Front. Psychol. 12:693396. doi: 10.3389/fpsyg.2021.693396, PMID: 34589021 PMC8475783

[ref14] Groarke J. M. Berry E. Graham-Wisener L. McKenna-Plumley P. E. McGlinchey E. Armour C. (2020). Loneliness in the UK during the COVID-19 pandemic: cross-sectional results from the COVID-19 psychological wellbeing study. PLoS One 15:e0239698. doi: 10.1371/journal.pone.0239698, PMID: 32970764 PMC7513993

[ref15] Huckins J. F. daSilva A. W. Wang W. Hedlund E. Rogers C. Nepal S. K.. (2020). Mental health and behavior of college students during the early phases of the COVID-19 pandemic: longitudinal smartphone and ecological momentary assessment study. J. Med. Internet Res. 22:e20185. doi: 10.2196/20185, PMID: 32519963 PMC7301687

[ref16] Islam A. Barna S. D. Raihan H. Khan N. A. Hossain M. T. (2020). Depression and anxiety among university students during the COVID-19 pandemic in Bangladesh: a web-based cross-sectional survey. PLoS One 15:e0238162. doi: 10.1371/journal.pone.0238162, PMID: 32845928 PMC7449469

[ref17] Khoshaim H. B. Al-Sukayt A. Chinna K. Nurunnabi M. Sundarasen S. Kamaludin K.. (2020). Anxiety level of university students during COVID-19 in Saudi Arabia. Front. Psych. 11:579750. doi: 10.3389/fpsyt.2020.579750, PMID: 33362601 PMC7759470

[ref19] Kroenke K. Strine T. W. Spitzer R. L. Williams J. B. W. Berry J. T. Mokdad A. H. (2009). The PHQ-8 as a measure of current depression in the general population. J. Affect. Disord. 114, 163–173. doi: 10.1016/j.jad.2008.06.026, PMID: 18752852

[ref20] Lee J. Solomon M. Stead T. Kwon B. Ganti L. (2021). Impact of COVID-19 on the mental health of US college students. BMC Psychol. 9:95. doi: 10.1186/s40359-021-00598-3, PMID: 34103081 PMC8185692

[ref21] Li Y. Wang A. Wu Y. Han N. Huang H. (2021). Impact of the COVID-19 pandemic on the mental health of college students: a systematic review and meta-analysis. Front. Psychol. 12:669119. doi: 10.3389/fpsyg.2021.669119, PMID: 34335381 PMC8316976

[ref22] Liu B. Huynh E. Li C. Wu Q. (2022). Impact of COVID-19 on college students at one of the most diverse campuses in the USA: a factor analysis of survey data. BMJ Open 12:e061719. doi: 10.1136/bmjopen-2022-061719, PMID: 36691145 PMC9445234

[ref23] Lv X. Ma J. Brinthaupt T. M. Zhao S. Ren X. (2022). Impacts of university lockdown during the coronavirus pandemic on college students’ academic achievement and critical thinking: a longitudinal study. Front. Psychol. 13:995784. doi: 10.3389/fpsyg.2022.995784, PMID: 36389610 PMC9643715

[ref24] Mack D. L. DaSilva A. W. Rogers C. Hedlund E. Murphy E. I. Vojdanovski V.. (2021). Mental health and behavior of college students during the COVID-19 pandemic: longitudinal Mobile smartphone and ecological momentary assessment study, part II. J. Med. Internet Res. 23:e28892. doi: 10.2196/28892 33900935 PMC8183598

[ref25] Newman A. Bavik Y. L. Mount M. Shao B. (2021). Data collection via online platforms: challenges and recommendations for future research. Appl. Psychol. 70, 1380–1402. doi: 10.1111/apps.12302

[ref26] Palan S. Schitter C. (2018). Prolific.Ac—a subject pool for online experiments. J. Behav. Exp. Financ. 17, 22–27. doi: 10.1016/j.jbef.2017.12.004

[ref27] Peer E. Rothschild D. Gordon A. Evernden Z. Damer E. (2021). Data quality of platforms and panels for online behavioral research. Behav. Res. Methods 54, 1643–1662. doi: 10.3758/s13428-021-01694-3, PMID: 34590289 PMC8480459

[ref29] Prowse R. Sherratt F. Abizaid A. Gabrys R. L. Hellemans K. G. C. Patterson Z. R.. (2021). Coping with the COVID-19 pandemic: examining gender differences in stress and mental health among university students. Front. Psych. 12:650759. doi: 10.3389/fpsyt.2021.650759, PMID: 33897499 PMC8058407

[ref31] Rettie H. Daniels J. (2021). Coping and tolerance of uncertainty: predictors and mediators of mental health during the COVID-19 pandemic. Am. Psychol. 76, 427–437. doi: 10.1037/amp0000710 32744841

[ref32] Sams N. Fisher D. M. Mata-Greve F. Johnson M. Pullmann M. D. Raue P. J.. (2021). Understanding psychological distress and protective factors amongst older adults during the COVID-19 pandemic. Am. J. Geriatr. Psychiatry 29, 881–894. doi: 10.1016/j.jagp.2021.03.005, PMID: 33867224 PMC8491780

[ref33] Spitzer R. L. Kroenke K. Williams J. B. W. Löwe B. (2006). A brief measure for assessing generalized anxiety disorder: the GAD-7. Arch. Intern. Med. 166, 1092–1097. doi: 10.1001/archinte.166.10.1092, PMID: 16717171

[ref34] Wang X. Chen H. Liu L. Liu Y. Zhang N. Sun Z.. (2020a). Anxiety and sleep problems of college students during the outbreak of COVID-19. Front. Psych. 11:588693. doi: 10.3389/fpsyt.2020.588693, PMID: 33329134 PMC7719633

[ref35] Wang X. Hegde S. Son C. Keller B. Smith A. Sasangohar F. (2020b). Investigating mental health of US College students during the COVID-19 pandemic: cross-sectional survey study. J. Med. Internet Res. 22:e22817. doi: 10.2196/22817, PMID: 32897868 PMC7505693

[ref36] Wathelet M. Duhem S. Vaiva G. Baubet T. Habran E. Veerapa E.. (2020). Factors associated with mental health disorders among university students in France confined during the COVID-19 pandemic. JAMA Netw. Open 3:e2025591. doi: 10.1001/jamanetworkopen.2020.25591, PMID: 33095252 PMC7584927

[ref37] Zheng C. Yi P. Shen G. Chen W. (2022). Effects of school resumption on college students’ mental health during the COVID-19 pandemic. J. Psychosoc. Nurs. Ment. Health Serv. 60, 19–27. doi: 10.3928/02793695-20211118-02 34846227

