# Supplementary material for: Mental health and academic experiences among U.S. college students during the COVID-19 pandemic
Source: Front Psychol. 2023 Apr 27;14:1166960. doi: 10.3389/fpsyg.2023.1166960 (PMC10176088; doi:10.3389/fpsyg.2023.1166960)
Supplement: Supplementary file 1 [file Table_1.DOCX]

Supplementary Material

Mental Health and Academic Experiences Among U.S. College Students During the COVID-19 Pandemic

Michael E. Roberts^*^, Elizabeth A. Bell, Jillian L. Meyer

*** Correspondence:** Corresponding Author [michaelroberts@depauw.edu](mailto:michaelroberts@depauw.edu)

# Supplementary Materials 1:

# Survey questions from fall 2020 study – a succinct version of the survey questions is below; a copy of the full survey and response options is available on this Google Form: <https://docs.google.com/forms/d/e/1FAIpQLSdqJVCMGgaq5d7_wdiVJAWwFgsNkNUjP1oY76X2tMDuGqkdOQ/viewform?usp=sf_link>

1. Has your perceived risk of contracting the COVID-19 virus changed since the Fall 2020 academic semester began (compared to before the semester)?

2. What is your current living situation during the Fall 2020 academic semester (select the option that best applies).

3. Rate your satisfaction with your current living situation on a scale from 1 to 5.

4. Has your mental health been impacted by your living situation during the Fall 2020 academic semester (compared to before the semester began)?

5. As you take classes during the Fall 2020 semester, which of these best describes where you live (compared to your campus)?

6. Do you have a quiet and comfortable place to do your classes and course work?

7. Which of the following best applies to your job status while taking classes during the Fall 2020 academic semester?

8. Are you currently/have you been in isolation or quarantine (under social isolation because you were diagnosed with COVID-19 or were a close contact of someone diagnosed with COVID-19)?

9. Since your fall 2020 academic semester began, how satisfied have you been with:

10. Since your fall 2020 academic semester began, how much support has been given to you?

11. Since your fall 2020 academic semester began, how much support and help have you given?

12. How much do you currently go out in public for essential reasons (going to classes, job, grocery, etc.) compared to pre-COVID?

13. How safe do you feel when going out in public for essential reasons (going to classes, job, grocery, etc.) compared to pre-COVID?

14. How much do you currently go out in public for non-essential reasons (e.g., shopping for non-essentials, eating at restaurants, entertainment, etc.) compared to pre-COVID?

15. How safe do you feel when going out in public for non-essential reasons (e.g., shopping for non-essentials, eating at restaurants, entertainment, etc.) compared to pre-COVID?

16. Responding thoughtfully to the questions is important for our study -- please choose 5

17. Estimate how many hours you spend on each of these media categories per day

18. Estimate how much you trust each category as an accurate source for your news

19. Rate how much each factor has recently contributed to your stress

20. How positive are your experiences in the following college class formats during the Fall 2020 academic semester (skip any college class formats that are not applicable for you)?

21. How strong is your sense of community in the following college class formats during the Fall 2020 academic semester (skip any college class formats that are not applicable for you)?

22. For this question, choose the number 2.

23. What is your confidence level in your ability to perform well academically during this Fall 2020 academic semester?

24. In the last month, how often have you been upset because of something that happened unexpectedly?

In the last month, how often have you felt that you were unable to control the important things in your life?

In the last month, how often have you felt nervous and “stressed”

In the last month, how often have you felt confident about your ability to handle your personal problems?

In the last month, how often have you felt that things were going your way?

In the last month, how often have you found that you could not cope with all the things that you had to do?

In the last month, how often have you been able to control irritations in your life?

In the last month, how often have you felt that you were on top of things?

In the last month, how often have you been angered because of things that were outside of your control?

In the last month, how often have you felt difficulties were piling up so high that you could not overcome them?

In the last month, we would like you to select 0 as an attention check for this question:

25. Over the last 2 weeks, how often have you been bothered by the following problems?

Feeling nervous, anxious or on

Not being able to stop or control worrying

Worrying too much about different things

Trouble relaxing

Being so restless that it is hard to sit still

Becoming easily annoyed or irritable

Feeling afraid as if something awful might happen

26. Over the last 2 weeks, how often have you been bothered by any of the following problems?

Little interest or pleasure in doing things

Feeling down, depressed, or hopeless

Trouble falling or staying asleep, or sleeping too much

Feeling tired or having little energy

Poor appetite or overeating

Not filling out this survey with accurate answers - select 0

Feeling bad about yourself — or that you are a failure or have let yourself or your family down

Trouble concentrating on things, such as reading the newspaper or watching television

Moving or speaking so slowly that other people could have noticed. Or the opposite — being so fidgety or restless that you have been moving around a lot more than usual

*Demographics*

Age (18-22, 23-30, 31-40, 41-50, 51 +)

Race/ethnicity -- Which of these categories best describes you? Select all that apply: (American Indian or Alaska Native, Asian or Asian-American, Black or African American, Hispanic, Latino, or Spanish Origin, Middle Eastern or North African, Native Hawaiian or Other Pacific Islander, White, Multi-Racial, Other)

Gender identity -- Which of these categories best describes you? (Female, Male, Non-binary, Other)

Class year in college (or equivalent in credits earned)? (First Year, Sophomore, Junior, Senior, Fifth-Year, Other)

In what country/continent are you living during the Fall 2020 academic semester?

If you are living in the United States during the Fall 2020 academic semester, which state?

Which of the following best describes the location you are living during the Fall 2020 academic semester (and this would be where your campus is located if you’re living on campus)? (Urban, Suburban, Rural (for our purposes, you can consider a small town to be rural, if it isn't a suburb of a large city))

What is your level of education?

# Supplementary Materials 2: Survey questions from spring 2021 study:

Survey questions from spring 2021 study – a succinct version of the survey questions is below; a copy of the full survey and response options is available on this Google Form: <https://docs.google.com/forms/d/e/1FAIpQLSevZj6XyWslE23lyLfMN5fVII0Zb9r_n3u_xHzOfRVmSFX0jA/viewform?usp=sf_link>

1. Rate on a scale of 1-5 how closely your university tracks COVID-19 cases on campus during the 𝐒𝐩𝐫𝐢𝐧𝐠 𝟐𝟎𝟐𝟏 semester.

2. Rate on a scale of 1-5 how much your university enforces mask-wearing.

3. Rate on a scale of 1-5 how much your university enforces social distancing.

4. Rate on a scale of 1-5 the effectiveness of your university’s COVID-19 testing policies for students during the 𝐒𝐩𝐫𝐢𝐧𝐠 𝟐𝟎𝟐𝟏 semester.

5. Rate on a scale of 1-5 how well your university respects your privacy on campus during the 𝐒𝐩𝐫𝐢𝐧𝐠 𝟐𝟎𝟐𝟏 semester.

6. Rate on a scale of 1-5 the quality of social life on your university’s campus during the 𝐒𝐩𝐫𝐢𝐧𝐠 𝟐𝟎𝟐𝟏 semester.

7. Rate on a scale of 1-5 how strict your university’s COVID-19 policies are in restricting student social behavior on campus during the 𝐒𝐩𝐫𝐢𝐧𝐠 𝟐𝟎𝟐𝟏 semester.

8. Rate on a scale of 1-5 how much social isolation (not because of university quarantine of individual students) is on your university campus.

9. Rate on a scale of 1-5 how well your university supports your choice to see friends (if you choose to do so).

10. Rate on a scale of 1-5 how well your university offers social programming (events, etc.) for students during the 𝐒𝐩𝐫𝐢𝐧𝐠 𝟐𝟎𝟐𝟏 semester.

11. Rate on a scale of 1-5 the quality of social life on your university’s campus during the 𝐒𝐩𝐫𝐢𝐧𝐠 𝟐𝟎𝟐𝟏 semester compared to a regular semester before the pandemic.

12. For this question, choose the number 2.

13. Rate on a scale of 1-5 how your university administration’s COVID-19 disciplinary responses have affected your stress levels.

14. Rate on a scale of 1-5 how safe you feel on your campus given the COVID-19 safety precautions put into place.

15. Rate on a scale of 1-5 how well your university communicates its COVID-19 safety policies during the 𝐒𝐩𝐫𝐢𝐧𝐠 𝟐𝟎𝟐𝟏 semester.

16. Rate on a scale of 1-5 whether the students you know tend to follow the university’s COVID-19 policies.

17. Did your university allow all students to return to campus during the 𝐒𝐩𝐫𝐢𝐧𝐠 𝟐𝟎𝟐𝟏 semester if they wanted to return?

18. Rate on a scale of 1-5 your satisfaction with your university’s COVID-19 response during the 𝐒𝐩𝐫𝐢𝐧𝐠 𝟐𝟎𝟐𝟏 semester.

19. Rate on a scale of 1-5 your satisfaction with your university’s COVID-19 response during the 𝐅𝐚𝐥𝐥 𝟐𝟎𝟐𝟎 semester.

20. In a typical semester before the pandemic began, how frequently did you take college courses online? (1 = None of my college courses were online before the pandemic, 5 = All of my college courses were online before the pandemic)

What is the class format for most of your classes during the 𝐒𝐩𝐫𝐢𝐧𝐠 𝟐𝟎𝟐𝟏 semester? (Fully online, Mix of in person and online, Fully in person)

22. Rate on a scale of 1-5 your satisfaction with your university’s academic experience (e.g., classes, training) during the 𝐒𝐩𝐫𝐢𝐧𝐠 𝟐𝟎𝟐𝟏 semester.

23. Rate on a scale of 1-5 your confidence level in your ability to perform well academically during this 𝐒𝐩𝐫𝐢𝐧𝐠 𝟐𝟎𝟐𝟏 academic semester.

24. Rate on a scale of 1-5 your confidence level in your ability to perform well academically during the 𝐅𝐚𝐥𝐥 𝟐𝟎𝟐𝟎 academic semester.

25. Rate on a scale of 1-5 your academic workload in your college courses during the 𝐒𝐩𝐫𝐢𝐧𝐠 𝟐𝟎𝟐𝟏 semester compared to taking college courses before the pandemic began.

26. Rate on a scale of 1-5 how understanding your academic instructors are of the need for accommodations related to your mental or physical health?

27. How positive are your experiences in the following college class formats (Fully online, Hybrid, In person) during the 𝐒𝐩𝐫𝐢𝐧𝐠 𝟐𝟎𝟐𝟏 academic semester?

28. How strong is your sense of community with your classmates in the following college class formats (Fully online, Hybrid, In person) during the 𝐒𝐩𝐫𝐢𝐧𝐠 𝟐𝟎𝟐𝟏 academic semester?

29. For this question, choose the number 2.

30. What is your current living situation during the 𝐒𝐩𝐫𝐢𝐧𝐠 𝟐𝟎𝟐𝟏 academic semester (select the option that best applies)? (Living on campus or within walking distance of campus, Living off campus within car commuting distance, Living off campus outside of commuting distance)

31. Rate on a scale of 1-5 your satisfaction with your current living situation.

32. What was your living situation during the 𝐅𝐚𝐥𝐥 𝟐𝟎𝟐𝟎 academic semester (select the option that best applies)? (Living on campus or within walking distance of campus, Living off campus within car commuting distance, Living off campus outside of commuting distance)

33. Rate on a scale of 1-5 your satisfaction with your living situation during the 𝐅𝐚𝐥𝐥 𝟐𝟎𝟐𝟎 semester.

34. Rate on a scale of 1-5 your satisfaction with your overall university experience (e.g., living circumstances, social life, university events, classes, etc.) during the 𝐒𝐩𝐫𝐢𝐧𝐠 𝟐𝟎𝟐𝟏 semester.

35. Rate on a scale of 1-5 your satisfaction with your sense of belonging in your campus community during the 𝐒𝐩𝐫𝐢𝐧𝐠 𝟐𝟎𝟐𝟏 semester.

36. Rate on a scale of 1-5 your satisfaction with the social support you are receiving (e.g. from friends, family, etc.) during the 𝐒𝐩𝐫𝐢𝐧𝐠 𝟐𝟎𝟐𝟏 semester.

37. Rate on a scale of 1-5 how often you have a quiet and comfortable place to do your course work and studying.

38. Rate on a scale of 1-5 how concerned you have been about financial issues during this 𝐒𝐩𝐫𝐢𝐧𝐠 𝟐𝟎𝟐𝟏 semester.

39. Rate on a scale of 1-5 how much your job or other non-academic work responsibilities have hindered your ability to focus on college.

40. Which of the following best applies to your job status while taking classes during the 𝐅𝐚𝐥𝐥 𝟐𝟎𝟐𝟎 academic semester? (Full time, Part-time, Furloughed, Looking for work, Not working, Other)

41. Has your perceived risk of contracting the COVID-19 virus changed since the 𝐒𝐩𝐫𝐢𝐧𝐠 𝟐𝟎𝟐𝟏 academic semester began (compared to before the semester)? (1-5)

42. Have you received a COVID-19 vaccination? (Yes, No)

43. If you have not received a COVID-19 vaccination, how enthusiastic are you about getting a vaccination? (1-5)

44. Responding thoughtfully to the questions is important for our study -- please choose 5

45. Estimate how many hours you spend on social media (Instagram, Twitter, Tiktok, Snapchat, and Facebook) per day (0 hours, 1 minute-59 minutes, 1-2 hours, 3-4 hours, 5+ hours)

46. Estimate how many hours you spend on news (Print and online newspapers, television, and streaming news shows) per day (0 hours, 1 minute-59 minutes, 1-2 hours, 3-4 hours, 5+ hours)

47. Are you currently/have been in isolation or quarantine? (Yes, I am in isolation or quarantine right now, Not right now, but I have been in isolation or quarantine, Not right now, but I have been in isolation or quarantine at some point since the Spring semester started, No, I have not been in isolation or quarantine, Other)

48. PSS Over the last 2 weeks, how often have you been bothered by any of the following problems?

In the last month, how often have you been upset because of something that happened unexpectedly?

In the last month, how often have you felt that you were unable to control the important things in your life?

In the last month, how often have you felt nervous and “stressed”

In the last month, how often have you felt confident about your ability to handle your personal problems?

In the last month, how often have you felt that things were going your way?

In the last month, how often have you found that you could not cope with all the things that you had to do?

49. PSS (cont.)

In the last month, how often have you been able to control irritations in your life?

In the last month, how often have you felt that you were on top of things?

In the last month, how often have you been angered because of things that were outside of your control?

In the last month, how often have you felt difficulties were piling up so high that you could not overcome them?

In the last month, we would like you to select 0 as an attention check for this question:

50. GAD Over the last 2 weeks, how often have you been bothered by the following problems?

Feeling nervous, anxious or on edge

Not being able to stop or control worrying

Worrying too much about different things

Trouble relaxing

Being so restless that it is hard to sit still

Becoming easily annoyed or irritable

Feeling afraid as if something awful might happen

51. PHQ Over the last 2 weeks, how often have you been bothered by any of the following problems?

Little interest or pleasure in doing things

Feeling down, depressed, or hopeless

Trouble falling or staying asleep, or sleeping too much

Feeling tired or having little energy

Poor appetite or overeating

51. PHQ (cont.)

Not filling out this survey with accurate answers - select 0

Feeling bad about yourself — or that you are a failure or have let yourself or your family down

Trouble concentrating on things, such as reading the newspaper or watching television

Moving or speaking so slowly that other people could have noticed. Or the opposite — being so fidgety or restless that you have been moving around a lot more than usual

Demographics

Age (18-22, 23-30, 31-40, 41-50, 51 +)

Race/ethnicity -- Which of these categories best describes you? Select all that apply: (American Indian or Alaska Native, Asian or Asian-American, Black or African American, Hispanic, Latino, or Spanish Origin, Middle Eastern or North African, Native Hawaiian or Other Pacific Islander, White, Multi-Racial, Other)

Gender identity -- Which of these categories best describes you? (Female, Male, Non-binary, Other)

Class year in college (or equivalent in credits earned)? (First Year, Sophomore, Junior, Senior, Fifth-Year, Other)

In what country/continent are you living during the 𝐒𝐩𝐫𝐢𝐧𝐠 𝟐𝟎𝟐𝟏 academic semester?

If you are living in the United States during the 𝐒𝐩𝐫𝐢𝐧𝐠 𝟐𝟎𝟐𝟏 academic semester, which state?

Which of the following best describes the location you are living during the 𝐒𝐩𝐫𝐢𝐧𝐠 𝟐𝟎𝟐𝟏 academic semester (and this would be where your campus is located if you’re living on campus)? (Urban, Suburban, Rural (for our purposes, you can consider a small town to be rural, if it isn't a suburb of a large city))

How many students attend your university? (Less than 5,000 students, 5,000-15,000 students, More than 15,000 students)

Is your college or university: (Public (for example, state universities), Private (for example, a small liberal arts college), Other)

Which of the following describes your total household income last year? (Less than $25,000, $25,000-50,000
